# Anti-Cancer Properties of Ginkgolic Acids in Human Nasopharyngeal Carcinoma CNE-2Z Cells via Inhibition of Heat Shock Protein 90

**DOI:** 10.3390/molecules26216575

**Published:** 2021-10-30

**Authors:** Hong-Mei Li, Hui Ma, Xiaolong Sun, Bohan Li, Chengjiang Cao, Yiqun Dai, Meilin Zhu, Cheng-Zhu Wu

**Affiliations:** School of Pharmacy, Bengbu Medical College, 2600 Donghai Road, Bengbu 233030, China; lihongmei@bbmc.edu.cn (H.-M.L.); mh9504@126.com (H.M.); sxl8172@126.com (X.S.); libohan1228@163.com (B.L.); b957667573@gmail.com (C.C.); daiyiqun25@126.com (Y.D.); zlyk521@126.com (M.Z.)

**Keywords:** ginkgolic acids, Hsp90, nasopharyngeal carcinoma, migration, invasion, apoptosis

## Abstract

*Ginkgo biloba* L. has been used in traditional Chinese medicine (TCM) for thousands of years. However, the anti-cancer properties of ginkgolic acids (GAS) isolated from *G. biloba* have not been investigated in human nasopharyngeal carcinoma cells. In this study, GAS exhibited an inhibitory effect on the ATPase activity of heat shock protein 90 (Hsp90) and anti-proliferative activities against four human cancer cell lines, with IC_50_ values ranging from 14.91 to 23.81 μg·mL^−1^. In vivo experiments confirmed that GAS inhibited tumor growth in CNE-2Z cell-xenografted nude mice with low hepatotoxicity. We further demonstrated that GAS suppressed migration and invasion and induced the apoptosis of CNE-2Z cells by inducing the degradation of Hsp90 client proteins (MMP-2, MMP-9, Her-2, c-Raf, Akt, and Bcl-2). Together, GAS are new Hsp90 inhibitors by binding to Hsp90 (hydrogen bond and hydrophobic interaction). Thus, GAS from *G. biloba* might represent promising Hsp90 inhibitors for the development of anti-nasopharyngeal carcinoma agents.

## 1. Introduction

In North Africa, Southeast Asia, and Southern China, nasopharyngeal carcinoma (NPC) has become the most frequent head and neck malignancy threatening human health [1,2]. Although most patients with local NPC respond well to radiotherapy/ chemotherapy, with an improved 5 year survival rate (approximately 80%), distant metastasis and local recurrence are still primarily responsible for treatment failure and death associated with NPC [3,4]. For these reasons, novel therapeutic drugs and approaches with improved outcomes for advanced NPC are needed.

Heat shock protein 90 (Hsp90) is emerging as an important target for the prevention and treatment of cancer, including NPC [5,6]. Hsp90 is an intracellular chaperone protein and is involved in the maturation and stabilization of more than 300 client proteins, such as Her-2, EGFR, Akt, c-Raf, HIF-1α, Bcr-Abl, CDK4, MMPs, and p53 [7,8,9]. Although Hsp90 is highly expressed in most cells, Hsp90 inhibitors display noteworthy selectivity for tumor cells compared with normal cells [10]. Currently, many Hsp90 inhibitors have been discovered and more than 10 agents are now in clinical trials for advanced cancer, such as 17-AAG, BIIB021, SNX-5422, PU-H71, and TAS-116 [11,12]. Previous research has shown that Hsp90 inhibitors exhibit significant anti-tumor properties in NPC, such as the inhibition of cell growth, induction of apoptosis, and suppression of invasion and metastasis [6,13,14,15]. Recently, we launched a screening program for Hsp90 inhibitors from traditional Chinese medicine (TCM), and identified ginkgolic acids (GAS) from *n*-hexane extracts of the seed coat of *Ginkgo biloba* L. 

*G. biloba* is a traditional medicine that has been widely used for thousands of years in China [16]. GAS exist in the leaves, nuts, and external seed coat of *G. biloba* L., including the monomers C15:1, C17:1, C13:0, C15:0, and C17:2 [17]. Many studies have reported that GAS exhibit various pharmacological activities, including anti-tumor, anti-depressant, anti-bacterial, anti-anxiety, and anti-viral activities [18,19,20,21,22]. Several previous studies have indicated that GAS can inhibit tumor invasion and migration, and induce apoptosis in several cancer types, including breast, lung, pancreatic, colon, and liver cancer cell lines [23,24,25,26,27]. However, the anti-tumor properties of GAS in NPC and their mechanism of action are not completely understood. In this novel study, we examined the anti-cancer effects of GAS on the NPC cell line CNE-2Z in vitro and in vivo. Furthermore, we investigated whether their anti-cancer properties could be associated with the inhibition of cell proliferation, the suppression of migration and invasion, and the induction of apoptosis in CNE-2Z cells, as well as the inhibition of Hsp90 chaperon function.

## 2. Results

### 2.1. GAS Inhibit Hsp90 ATPase Activity 

To understand the in vitro anti-cancer activity of GAS, we tested their ability to inhibit Hsp90 ATPase activity using a malachite green assay. Geldanamycin (GM) was used as the positive control. As shown in Table 1, GAS and GA (15:1) both inhibited the Hsp90 ATPase activity, with an IC_50_ value of 32.76 μg·mL^−1^ and 69.65 μM, respectively. 

### 2.2. GAS Exhibit an Anti-Proliferative Activity in CNE-2Z Cells

**Figure 1:**
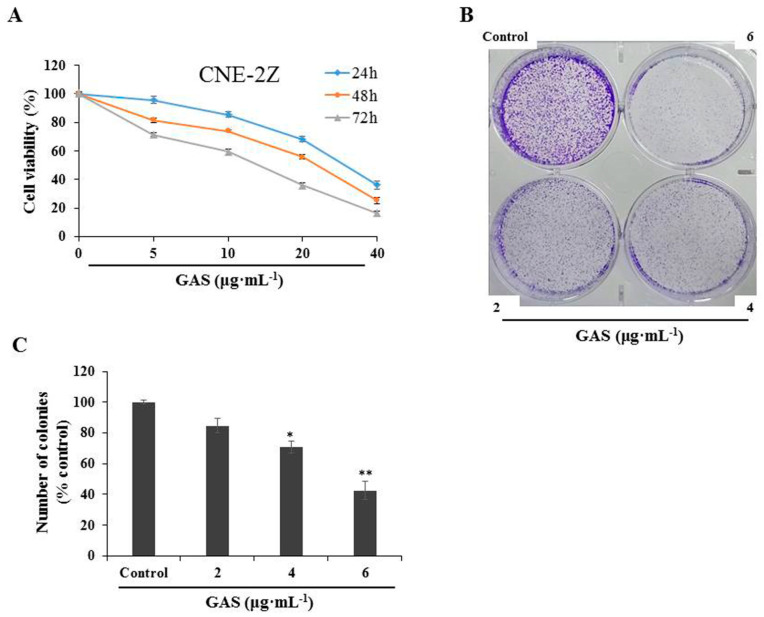
Inhibitory role of GAS on CNE-2Z cells. (**A**) CNE-2Z cells were treated with increasing concentrations (0, 5, 10, 20, and 40 μg·mL^−1^) of GAS for 24, 48, and 72 h. Cell viability was measured through an MTT assay. (**B**) The colony forming capability of CNE-2Z cells was measured using a colony formation assay after being treated with low concentrations (0, 2, 4, and 6 μg·mL^−1^) of GAS for 5 days. (**C**) Quantification of the colony-forming capability of CNE-2Z cells inhibited by GAS. * *p* < 0.05 and ** *p* < 0.01 compared with the control.

GAS were evaluated for cytotoxicity through MTT assays on four human cancer cells. Preliminary screening revealed that the highest inhibitory activity of GAS on cell viability was exhibited in CNE-2Z cells, with an IC_50_ value of 14.91 μg·mL^−1^ at 72 h after treatment (Table 2). To investigate the in vitro effect of GAS treatment on human nasopharyngeal carcinoma, CNE-2Z cells were treated with various concentrations of GAS for 24, 48, and 72 h. As shown in Figure 1A, GAS significantly inhibited the proliferation of CNE-2Z cells in a concentration- and time-dependent manner. Similarly, the colony-formation assay also revealed an anti-proliferative activity of GAS at low concentrations (Figure 1B,C). 

### 2.3. In Vivo Anti-Tumor Efficacy of GAS 

**Figure 2:**
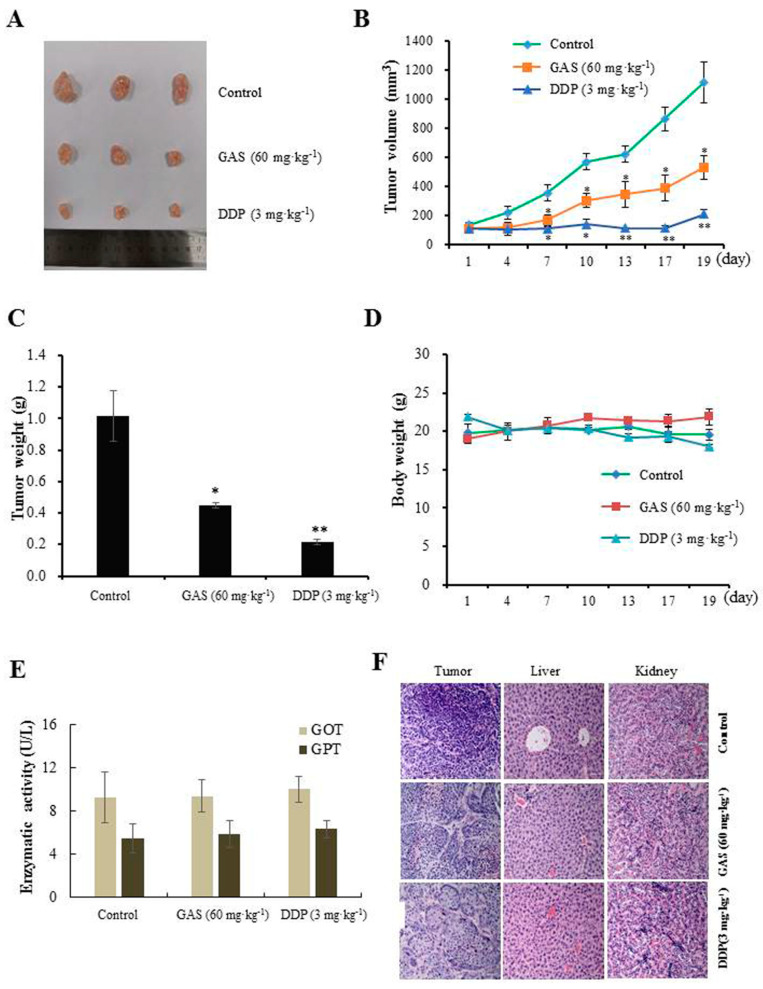
Anti-tumor efficacy of GAS on CNE-2Z cells xenograft in nude mice (*n* = 3). (**A**) Representative images of subcutaneous xenograft tumors from each treatment group. (**B**) Measurements of tumor volume. (**C**) Measurements of tumor weight after treatment for 19 days. (**D**) Body weight of mice. (**E**) Serum GOT and GPT levels as determined using an assay kit. (**F**) H&E staining of tumors, livers, and kidneys from mice after treatment (magnification: 200×). * *p* < 0.05 and ** *p* < 0.01 compared to the control.

To test the anti-tumor efficacy of GAS in NPC development, CNE-2Z cells were xenografted into nude mice, and then the mice were treated with GAS (60 mg·kg^−1^) for 19 days by intraperitoneal injection. As expected, GAS significantly suppressed tumor growth and shrank the tumor volume to 529.4 mm^3^ compared with 1118.2 mm^3^ in the control group, accounting for a 52.7% decrease in tumor volume (Figure 2A–C). However, no significant body weight loss was observed (Figure 2D). Additionally, GOT and GPT levels were not significantly altered by treatment with GAS compared with the control and DDP groups (Figure 2E). H&E staining results showed that GAS did not damage organs such as the liver and kidney (Figure 2F). These results indicated that GAS might potentially be used as anti-tumor agents with low toxicity in vivo. 

### 2.4. GAS Suppress Migration and Invasion of CNE-2Z Cells

**Figure 3:**
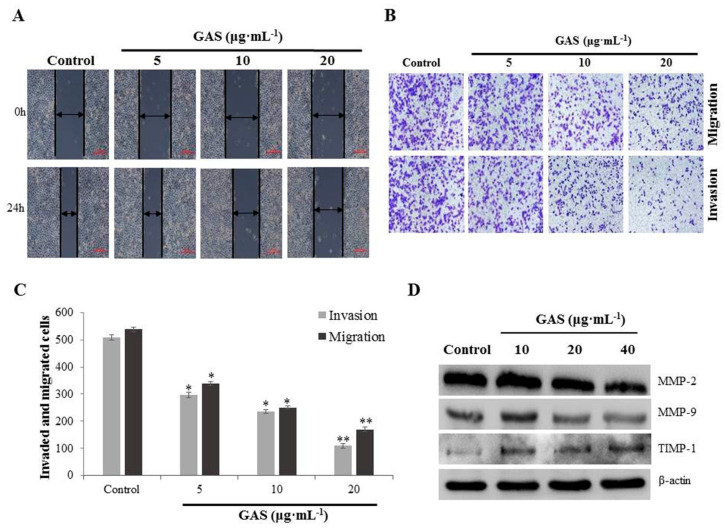
GAS suppressed invasion and migration of CNE-2Z cells. (**A**) Migration was analyzed by a wound-healing assay after being treated with various concentrations (5, 10, and 20 μg·mL^−1^) of GAS. (**B**) Cell invasion and migration were determined using a transwell assay after treatment with GAS (5, 10, and 20 μg·mL^−1^). (**C**) Quantification of invasion and migration of CNE-2Z cells suppressed by GAS. (**D**) Western blotting analyses of MMP-2, MMP-9, and TIMP-1 levels in CNE-2Z cells. β-Actin was used as an internal control. * *p* < 0.05 and ** *p* < 0.01 compared to the control.

Tumor invasion and metastasis are the main causes of treatment failure and high mortality in most cancer patients, including those with NPC. Thus, the effect of GAS on the migration and invasion of CNE-2Z cells was investigated. Wound-healing assays showed that GAS significantly suppressed the migration of CNE-2Z cells compared with the control group (Figure 3A). Moreover, transwell assays were performed to investigate the effect of GAS on migration and invasion ability. As shown in Figure 3B–C, 5 μg·mL^−1^ GAS treatment significantly reduced the number of CNE-2Z cells that crossed the membrane of the transwell chamber compared with the control (*p* < 0.05). The results also showed that GAS suppressed the migration and invasion of CNE-2Z cells in a concentration-dependent manner. Western blotting results showed that matrix metalloproteinase-2 (MMP-2) and MMP-9 expression were significantly down-regulated with increasing concentrations of GAS, while TIMP-1 protein expression gradually increased (Figure 3D and Figure S1). 

### 2.5. GAS Induce Apoptosis in CNE-2Z Cells

**Figure 4:**
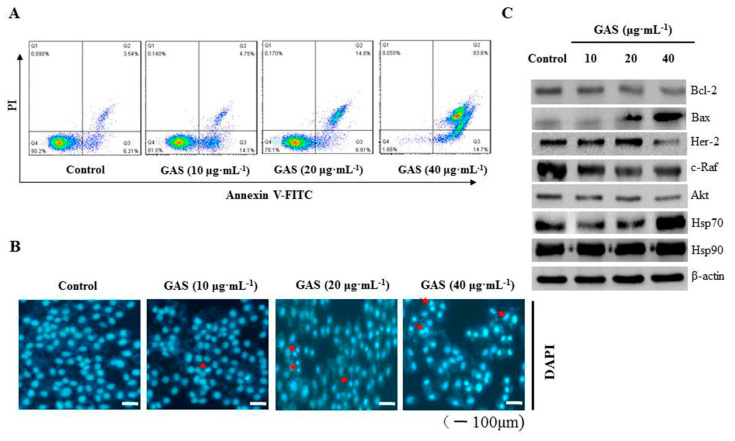
GAS induction of apoptosis in CNE-2Z cells. (**A**) Flow cytometric analysis of cell death after being treated with GAS using Annexin V-FITC/PI staining. (**B**) CNE-2Z cells treated with GAS for 24 h, subjected to DAPI staining, and visualized using fluorescence microscopy. Red arrowheads indicate apoptotic cells. (**C**) Western blot analyses of apoptosis-related protein levels in CNE-2Z cells. β-actin was used as an internal control.

We further investigated if the effect of GAS on CNE-2Z cell death involved apoptosis by using flow cytometry. As shown in Figure 4A, GAS significantly induced CNE-2Z cell apoptosis in a concentration-dependent manner. Moreover, DAPI staining revealed that specific morphological characteristics of apoptosis, such as bright nuclear condensation, occurred after increasing the concentration of GAS (Figure 4B). Western blotting results showed that GAS induced the degradation of Hsp90 client proteins (Her-2, c-Raf, Akt, and Bcl-2) and increased the expression of Bax and Hsp70, whereas it did not affect Hsp90 expression in CNE-2Z cells (Figure 4C and Figure S2). These findings indicate that GAS can induce apoptosis of CNE-2Z cells via the inhibition of Hsp90 client proteins.

### 2.6. Molecular Docking of GA (15:1) on Hsp90 

**Figure 5:**
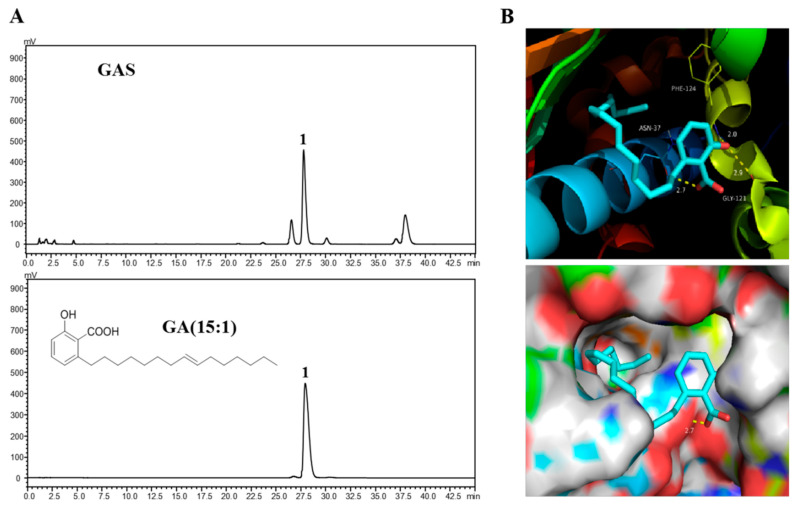
Molecular docking model of GA (15:1) with Hsp90α. (**A**) Chemical structure of GA (15:1). (**B**) Docking position of GA (15:1) in the active site of the Hsp90α N-terminal. Key amino acid residues within the binding site are indicated using capped sticks.

As shown in Figure 5A, one major peak was identified as GA (15:1, peak **1**), in which GA (15:1) accounted for 48.04%. This result is consistent with the findings of Jiang et al., who reported that the major component in the n-hexane extracts of *G. biloba* L. seeds was GA (15:1) [17]. In this study, the binding mode of GA (15:1) was revealed based on docking (Figure 5B). The carboxyl moiety on the aromatic ring formed a hydrogen bond with the residue Asn37. Meanwhile, the phenolic hydroxyl moiety of GA (15:1) formed hydrogen bonds with Gly121 and Phe124. Moreover, a hydrophobic interaction was formed between the alkyl chain moiety to occupy the lipophilic Hsp90α pocket.

## 3. Discussion

Certain compounds used in TCM have potential anti-cancer properties and low toxicity, improving quality of life, side effects, and therapeutic effects [28,29]. Thus, the discovery and development of anti-cancer drugs with low toxicity and high efficiency from TCM has become a popular subject in recent years. Recently, Hsp90 has been recognized as a promising biomarker for NPC screening, including at the early stage [14]. The Hsp90 expression was shown to be higher in NPC tissue than in non-cancerous nasopharyngeal mucosa tissue, and Hsp90 inhibitors have been shown to exhibit significant anti-tumor properties on NPC [6]. Therefore, Hsp90 inhibitors could show selectivity towards NPC cells, yielding specific anti-cancer effects. 

Consequently, we previously performed primary screening of 324 methanol extracts from TCM to identify Hsp90 inhibitors. Among them, we identified GAS from *n*-hexane extracts of the seed coat of *G. biloba*. The anti-cancer effect of GAS has been reported in several cancers in vitro and in vivo, but not in NPC. Here, we show that GAS are effective inhibitors of Hsp90 ATPase activity and demonstrate their anti-proliferative activities against NPC in vitro and in vivo, with low toxicity. We observed that GAS had a better inhibitory effect on the proliferation of CNE-2Z cells than the other cell lines (including MCF-7, MDA-MB-231, and H1975 cells). 

Unlike other head and neck cancers, NPC features the early invasion of surrounding tissues and metastasis [30]. Thus, inhibition of NPC cell invasion and metastasis may be of great clinical significance in improving the survival of NPC patients. In this study, GAS significantly suppressed the migration and invasive ability of CNE-2Z cells in vitro, suggesting that GAS suppress the invasion and metastasis of NPC. MMP-2 and MMP-9 play major roles in cancer cell invasion and metastasis [31]. Moreover, extracellular Hsp90 promotes cell motility, invasion, and metastasis in an MMP-2/9-dependent manner [32]. In the present study, we found that MMP-2 and MMP-9 were significantly down-regulated, while TIMP-1 was up-regulated in NPC cells following GAS treatment. Thus, GAS inhibited cell migration and invasion by inhibiting Hsp90 chaperon functions in CNE-2Z cells. 

Apoptosis is a programmed cell death (PCD) that plays a major role in the killing of cancer cells [33]. Flow cytometry and DAPI staining results indicated that GAS significantly induced the apoptosis of CNE-2Z cells in a concentration-dependent manner. The anti-apoptotic molecule Bcl-2 prevents apoptosis by suppressing the activity of Bax, which is an important pro-apoptotic molecule [34]. GAS treatment resulted in a reduced Bcl-2/Bax ratio, which suggests that Bcl-2 participates in GAS-promoted apoptosis of CNE-2Z cells. In addition, Western blotting also indicated that several key Hsp90 client protein (Her-2, c-Raf, and Akt) levels were down-regulated by GAS in CNE-2Z cells. 

In the present study, we observed that GAS had no significant effect on Hsp90 expression, which suggests that GAS inhibited Hsp90 ATPase activity and had no effect on protein expression. Several studies have reported that the activation of Hsp90 by phosphorylation is very important [35,36,37]. The phosphorylation of Hsp90 governs its interactions with co-chaperones and client proteins and could affect its stability. Unfortunately, we were unable to detect the phosphorylation of Hsp90 levels.

The seed coats of *G. biloba* are traditionally considered toxic, so the utilization rate of the *G. biloba* exopleura remains low [38,39]. However, our study shows that the *n*-hexane extracts of seed coats of *G. biloba* are effective against NPC cell lines through the inhibition of Hsp90 ATPase activity, while having low toxicity. A previous study has verified that GAS constitute a main group of components from the seed coats of *G. biloba*, including the monomers C15:1 (45%), C17:1 (38.5%), C13:0 (11.5%), C15:0 (3%), and C17:2 (2%) [17]. Here, we used GA (15:1) for docking with the target protein to indirectly reflect the binding affinity of GAS with Hsp90. The binding energy of Hsp90 toward GA (15:1) was -9.8 kcal/mol, indicating that it possessed strong binding affinity through hydrogen bonds and hydrophobic interactions. The strong interaction of GA (15:1) with Hsp90 supports the role of GAS as an Hsp90 inhibitor.

## 4. Materials and Methods

### 4.1. Plant Material

The *G. biloba* L. seeds were collected in October 2018 from a farm located in Bengbu city, and the authenticity of the plant was confirmed by Prof. Xian Li (School of Pharmacy, Bengbu Medical College). The GAS extract was prepared from the seed coat of *G. biloba*, as described previously [40].

### 4.2. Regents

GA (15:1) was purchased from the National Institutes for Food and Drug Control. Dulbecco’s modified Eagle medium (DMEM), RPMI-1640 medium, and trypsin were purchased from Hyclone (UT, USA). Fetal bovine serum (FBS) was purchased from Sijiqing Biotechnology (Hangzhou, China). Crystal violet and an annexin V-FITC apoptosis detection kit were purchased from Beyotime Biotechnology (Shanghai, China). Dimethyl sulfoxide (DMSO), 3-(4,5-dimethyl thiazol-2-yl)-2,5-diphenyl tetrazolium bromide (MTT) and 4,6-diamidino-2-phenyl indole (DAPI) were purchased from Sigma-Aldrich (St. Louis, MO, USA). Transwell inserts were purchased from Corning Life Sciences (NY, USA). Matrigel was obtained from BD Biosciences (Bedford, MA, USA). The antibody Anti-Hsp90 was purchased from Santa Cruz Biotechnology (Santa Cruz, CA, USA). Anti-Hsp70 was purchased from Bioss Biotechnology (Beijing, China). Anti-MMP-9, anti-MMP-2, and anti-Bcl-2 were purchased from Proteintech Group (Chicago, USA). Anti-Bax, anti-Her-2, anti-c-Raf, and anti-Akt were purchased from Cell Signaling Technology, Inc. (Danvers, MA, USA). Anti-TIMP-1 was purchased from Boster Biotechnology (Wuhan, China). Anti-β-actin was purchased from Abbkine Scientific (California, USA). The secondary antibodies goat anti-rabbit IgG and rabbit anti-mouse IgG were purchased from BioSharp Life Sciences (Hefei, China). The glutamic-oxaloacetic transaminase (GOT) and glutamic-pyruvic transaminase (GPT) detection kit were purchased from Jiancheng Bioengineering Institute (Nanjing, China). 

### 4.3. Determination of Hsp90 ATPase Activity

Hsp90 ATPase activity was measured using the malachite green assay, as described previously [41]. Briefly, 2 μM Hsp90 protein, 0.2 mM ATP, and samples were added to 96-well plates and incubated for 2.5 h at 37 °C. After incubation, 80 μL of malachite green-molybdate solution was added to each well, and the plate was shaken, followed by quenching with 34% sodium citrate. The absorbance was measured at 620 nm using a microplate reader (Synergy HT, BioTek, USA). 

### 4.4. Cell Lines and Cell Cultures

The human nasopharyngeal carcinoma cell line (CNE-2Z), breast cancer cell lines (MD-MB-231 and MCF-7), and lung cancer cell line (H1975) were obtained from the Chinese Academy of Sciences Cell Bank (Shanghai, China). Cells were cultured in DMEM or RPMI-1640 supplemented with 10% FBS and 1% penicillin/streptomycin. Cell culture was conducted in an incubator at 37°C and 5% CO_2_. 

### 4.5. Determination of Cell Viability 

Cells were incubated in 96-well plates at a density of 5 × 10^3^ cells per well and cultured overnight. After adherence, the cells were treated with varying concentrations (0, 5, 10, 20, and 40 μg·mL^−1^) of GAS for 24, 48, and 72 h. After the mentioned time points, the cell viability was examined using a standard MTT assay. GM was used as a positive control.

### 4.6. Colony-Formation Assay

CNE-2Z cells were seeded in 6-well plates at 4 × 10^3^ cells per well and cultured overnight. When colonies formed, the medium was replaced with fresh medium containing GAS at various concentrations (0, 2, 4, and 6 μg·mL^−1^), and cells were cultured for six days. After 6 days, the cells were washed with PBS and fixed with 4% paraformaldehyde for 10 min, followed by being stained with crystal violet for 10 min and photographed.

### 4.7. In Vivo Anti-Tumor Experiments

CNE-2Z cells (5 × 10^6^ cells per mouse) were subcutaneously injected into the right back of mice (female BALB/c nude mice, 4–5 weeks of age, obtained from Canes Laboratory, Changzhou, China). All experimental procedures were performed in accordance with the NIH guidelines for Care and Use of Laboratory Animals; all procedures and protocols were approved by the Animal Care and Use Committee of Bengbu Medical College. When approximately 100 mm^3^ of tumor volume was measured, mice were randomized in three groups (3 mice per group). Cis-platinum (DDP, 3 mg·kg^−1^) was used as a positive control drug. GAS (60 mg·kg^−1^) were intraperitoneally injected every 3 days for 19 days. The tumor volume (calculated from tumor length × width^2^/2) and body weights were determined every 3 days. After treatment for 19 days, the mice were sacrificed and serum GOT and GPT levers were determined. In addition, the solid tumors, livers, and kidneys were removed and preserved in a 4% formalin solution, and then stained with hematoxylin and eosin (H&E).

### 4.8. Wound-Healing Assay

CNE-2Z cells were seeded in 6-well plates precoated with 0.1% gelatin. After scraping the cell monolayer with a 10 μL pipet tip, the wells were washed twice with serum-free DMEM medium, replaced with serum-free medium containing various concentrations (0, 5, 10, and 20 μg·mL^−1^) of GAS, and then incubated for 24 h. The image of each scratch was acquired at 0 h and 24 h. Migration was assessed and imaged at the same location, and the healed area was measured. 

### 4.9. Cell Migration Assay

Migration was assessed using transwell chambers equipped with 8.0 μm pore membrane inserts without matrigel. CNE-2Z cells (5 × 10^4^/well) were added to the upper chambers and incubated for 24 h following treatment with different concentrations (0, 5, 10, and 20 μg·mL^−1^) of GAS. The lower chambers were filled with 10% FBS as the chemoattractant under normoxic conditions. After 24 h, the cells that had migrated were stained with 0.1% crystal violet and photographed under a light microscope at 200× magnification. The number of migratory cells was counted and analyzed to determine statistically significant differences. The experiments were performed independently three times. 

### 4.10. Cell Invasion Assay

The transwell invasion assay was performed using a 24-well plate with 8.0 μm pore membrane inserts that were coated with 50 μL of matrigel. CNE-2Z cells (5 × 10^4^/well) were added to the upper wells and incubated with different concentrations (0, 5, 10, and 20 μg·mL^−1^) of GAS for 36 h. After 36 h under normoxic conditions, the cells that invaded were stained with crystal violet and photographed under a light microscope at 200 × magnification. The number of invasive cells was counted and analyzed to determine statistically significant differences. The experiments were performed independently three times. 

### 4.11. Western Blotting

CNE-2Z cells were seeded in 6-well plates at a density of 5 × 10^5^ cells/well. After treatment with GAS, the cells were harvested, washed twice with PBS, and lysed using RIPA buffer for 30 min on ice. After centrifugation at 15,000 rpm for 30 min, the lysates were loaded onto 10% sodium dodecyl sulfate polyacrylamide gel electrophoresis (SDS-PAGE) and transferred to polyvinylidene fluoride (PVDF) membranes. The membrane was blocked with 5% skimmed milk and incubated with primary antibodies (MMP-2, MMP-9, TIMP-1, Bcl-2, Bax, Her-2, c-Raf, Akt, Hsp70, and Hsp90) overnight at 4°C. After washing with TBST buffer, the membranes were incubated with secondary antibodies for 2 h at room temperature. Next, protein bands were visualized using a chemiluminescence kit and detected using a gel imaging system (Bio-Rad, USA). Anti-β-actin was used as an internal control.

### 4.12. Flow Cytometry with Annexin V/ PI Staining

CNE-2Z cells were seeded in a 12-well plate at 2.5 × 10^5^ cells per well, as described above. After 24 h, the cells were harvested, washed with PBS, and stained with Annexin V-FITC solution, followed by the addition of propidium iodide (PI) staining solution and subsequent incubation according to the manufacturer’s protocol. The percentage of apoptotic cells was measured by Accuri C6 flow cytometry (BD Biosciences, State of New Jersey, USA). 

### 4.13. DAPI Staining

DAPI staining was used to determine apoptotic cells. CNE-2Z cells (2.5 × 10^5^/ well) were seeded in a 6-well plate and incubated overnight. After adherence, the cells were treated with various concentrations (0, 10, 20, and 40 μg·mL^−1^) of GAS. After 48 h, the cells were washed with PBS, fixed with 4% paraformaldehyde for 15 min, and then stained with DAPI solution for 5 min in the dark. Nuclear morphology was imaged using a fluorescence microscope (Olympus, Tokyo, Japan). 

### 4.14. Molecular Docking

PyRx was used for molecular modeling and docking studies. The crystal structure of GA (15:1) bound to Hsp90α (PDB code: 2XX2) was utilized for modeling experiments via Autodock Vina. PyMOL was used for further visualization, figure preparation, and conformation analysis. 

### 4.15. Statistical Analysis

Data are presented as the mean ± standard deviation (SD) from three independent experiments. One-way analysis of variance (ANOVA) followed by Dunnett’s post hoc test for multiple comparisons were used for statistical analysis of the data using version 16.0 of the SPSS software. * *p* < 0.05 and ** *p* < 0.01 were considered statistically significant.

## 5. Conclusions

The findings of this study demonstrate that GAS extracted from the seed coats of *G. biloba* show in vitro Hsp90 ATPase inhibitory activity and cytotoxic activity against four human cancer cell lines. In addition, GAS exhibit anti-cancer properties, affecting the proliferation, migration, invasion, and apoptosis of CNE-2Z cells in vitro and in vivo. Thus, GAS and related alkylphenols might potentially be used as novel Hsp90 inhibitors for the development of anti-cancer treatments against NPC.

## Figures and Tables

**Table 1:** Effect of ginkgolic acids on yeast Hsp90 ATPase inhibition using a malachite green assay.

Compounds	Yeast Hsp90 ATPase (IC_50_)
GAS (μg·mL^−1^)	32.76 ± 0.53
GA (15:1) (μM)	69.65 ± 1.02
GM (μM)	3.11 ± 0.21

**Table 2:** Anti-proliferative activities (IC_50_) of GAS in four human cancer cells.

Compounds	CNE-2Z	MCF-7	MDA-MB-231	H1975
GAS ( μg·mL^−1^)	14.91 ± 1.03	23.81 ± 0.81	23.11 ± 0.42	17.52 ± 0.52
GM (μM) ^a^	0.34 ± 0.05	0.19 ± 0.03	0.31 ± 0.02	0.41 ± 0.06

^a^ GM was used as the positive control.

## Data Availability

The data generated and analyzed during the study are available in this article.

## Supplementary Materials

The following are available online. Figure S1: Effects of GAS on expression level of MMP-2, MMP-9, and TIMP-1 in CNE-2Z cells by western blotting analysis, Figure S2: Effects of GAS on expression level of Bcl-2, Bax, Her-2, c-Raf, Akt, Hsp70, and Hsp90 in CNE-2Z cells by western blotting analysis.

## Sample Availability

Samples of the GAS and GA (15:1) are available from the authors.
