# Supplementary material for: Anti-Cancer Properties of Ginkgolic Acids in Human Nasopharyngeal Carcinoma CNE-2Z Cells via Inhibition of Heat Shock Protein 90
Source: Molecules. 2021 Oct 30;26(21):6575. doi: 10.3390/molecules26216575 (PMC8588116; doi:10.3390/molecules26216575)
Supplement: Supplementary file 1 [file molecules-26-06575-s001.zip › molecules-1416640-supplementary.pdf]

Supplementary material for

Article

Anti-cancer properties of ginkgolic acids in human nasopharyngeal carcinoma CNE-2Z cells via inhibition of heat shock protein 90

Hong-Mei Li[1, †], Hui Ma[1, †], Xiaolong Sun[1], Bohan Li[1], Chengjiang Cao[1], Yiqun Dai[1] , Meilin Zhu[1], Cheng-Zhu Wu[1, *]

[1] School of Pharmacy, Bengbu Medical College, 2600 Donghai Road, Bengbu 233030, Anhui, China;

lihongmei@bbmc.edu.cn (H.-M.L.); mh9504@126.com (H.M.); sxl8172@126.com (X.S.); libohan1228@163.com (B.L.); b957667573@gmail.com (C.C.); daiyiqun25@126.com (Y.D.); zlyk521@126.com (M.Z.)

* Correspondence: wuchengzhu0611@bbmc.edu.cn (C.-Z.W.) ;Tel.: +86-552-317-5232

[†] These authors contributed equally to this work.

Figure S1 Effects of GAS on expression level of MMP-2, MMP-9, and TIMP-1 in CNE-2Z cells by western blotting analysis 3

Figure S2 Effects of GAS on expression level of Bcl-2, Bax, Her-2, c-Raf, Akt, Hsp70, and Hsp90 in CNE-2Z cells by western blotting analysis_.. 4

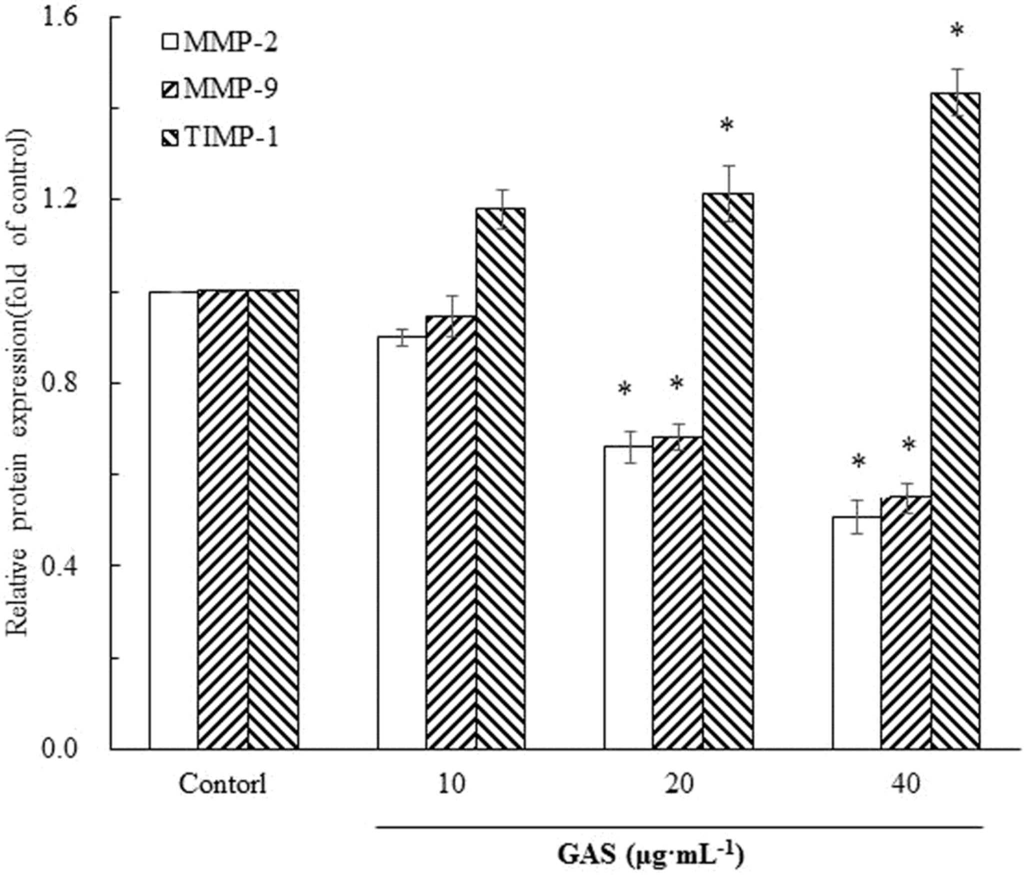

Figure 1S. Effects of GAS on expression level of MMP-2, MMP-9, and TIMP-1 in CNE-2Z cells by western blotting analysis. ($\bar{x} \pm s$, n=3), *p<0.05 compared with control.

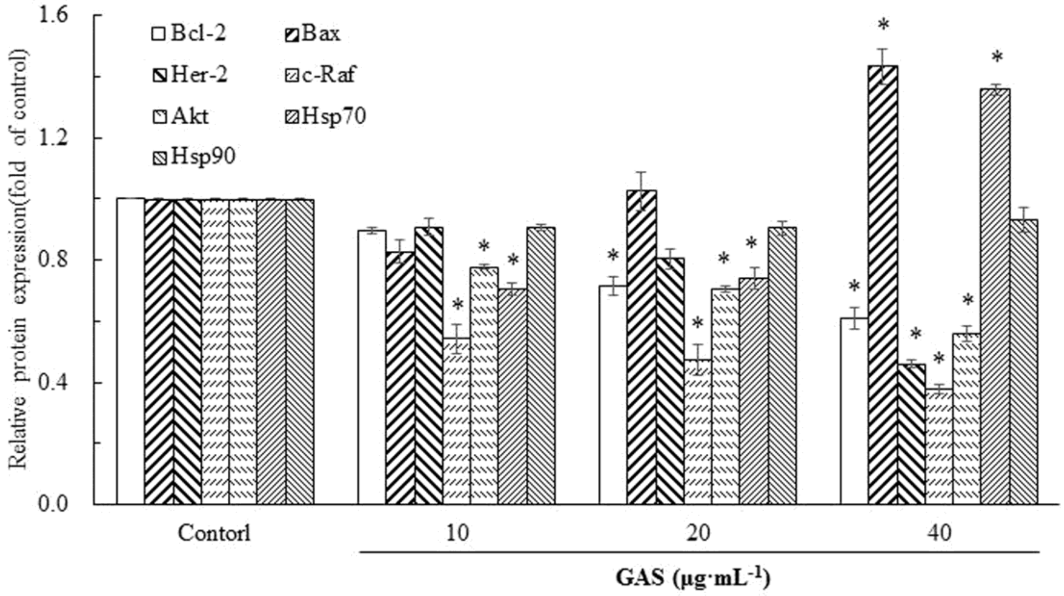

Figure 2S. Effects of GAS on expression level of Bcl-2, Bax, Her-2, c-Raf, Akt, Hsp70, and Hsp90 in CNE-2Z cells by western blotting analysis. ($\bar{x} \pm s$, n=3), *p<0.05 compared with control.
